# Editorial: Inflammaging and immunosenescence: role in aging-associated cardiovascular diseases

**DOI:** 10.3389/fcvm.2025.1616623

**Published:** 2025-05-14

**Authors:** Tlili Barhoumi, Rihab Bouchareb, Fayhan Alroqi, Adel Nefzi, Stephen Todryk

**Affiliations:** ^1^ Medical Research Core Facility and Platforms, King Abdullah International Medical Research Center (KAIMRC), King Saud Bin Abdulaziz University for Health Sciences (KSAU-HS), Ministry of National Guard Health Affairs, Riyadh, Saudi Arabia; ^2^ Department of Cardiovascular Sciences, Center for Metabolic Disease Research (CMDR), Temple University Lewis Katz School of Medicine (LKSM), Philadelphia, PA, United States; ^3^ Department of Pediatric, King Abdullah Specialized Children’s Hospital (KASCH), Ministry of the National Guard—Health Affairs, Riyadh, Saudi Arabia; ^4^ College of Medicine, King Saud Bin Abdulaziz University for Health Sciences (KSAU-HS), Riyadh, Saudi Arabia; ^5^ Herbert Wertheim College of Medicine. Center for Translational Science, Florida International University, Miami, FL, United States; ^6^ Faculty of Health & Life Sciences, Northumbria University, Newcastle upon Tyne, United Kingdom; ^7^ Translational & Clinical Research Institute, Newcastle University, Newcastle upon Tyne, United Kingdom

**Keywords:** inflammaging, immunosenescence, cardiovascular, elderly, vascular senescence, dietary antioxidant

**Editorial on the Research Topic**
Inflammaging and immunosenescence: role in aging-associated cardiovascular diseases

Aging is associated with chronic low-grade inflammation, known as “inflammaging”, and a decline in the function of the immune system, known as “immunosenescence”, both of which drive age-related disruption of molecular and cellular homeostasis leading to a range of diseases. Increasingly, research has pointed to immune system dysfunction and chronic inflammation as key drivers of vascular, cardiac, renal, and hepatic injury, or even dysregulation of the post-viral immune response leading to cardiovascular disease (CVD) pathogenesis. Although inflammaging and immunosenescence have been established as central modulators of aging-associated cardiovascular disease progression and complications, precise characterization of this health condition in elderly individuals is still challenging, and more studies in this area are needed to clarify biological pathways involving inflammaging and immunosenescence in different organs. This editorial summarizes new findings from recent studies including small vessel, pulmonary artery, dietary antioxidant, and long COVID, which suggest the pivotal role of inflammaging and immunosenescence in aging-associated CVD.

Müller and Di Benedetto, in a review described the process of interaction between immunosenescence and inflammaging, which drives cardiovascular disease through activation of NF-κB and NLRP3 inflammasome pathways, triggering release of IL-1β, IL-6 and IL-18 pro-inflammatory cytokines and production of oxidative stress, promoting vascular damage and atherosclerosis. This inflammatory condition is further exacerbated by immune cell (macrophages, *T* cells) dysfunction and the release of mitochondrial DNA fragments [Damage-associated molecular patterns (DAMPs)] and Reactive Oxygen Species (ROS). The authors explained the interaction of inflammation and decline in immune function in the elderly, creating a vicious cycle process that begins with immunosenescence, inducing persistence of inflammation which in turn accelerates immune dysfunction leading to increased risk of CVD. Furthermore, these effects disturbed cellular damage repair and antioxidant processes. The authors highlighted emerging research describing long-term cardiovascular complications (heart failure, stroke, arrhythmias) associated with COVID-19 infection in elderly subjects, characterized by fibrosis and vascular damage, suggesting an exaggeration of the pathologic condition due to immunosenescence and inflammaging. They concluded their review by highlighting the urgent need for research to develop strategies to alleviate vascular dysfunction in vulnerable elderly populations and promote healthier aging.

Small vessel endothelial dysfunction triggers vascular injury, a precursor to CVD, which is a major cause of mortality and comorbidity in elderly individuals.

A review by Kasal et al., described the interaction between microvascular endothelial dysfunction and inflammation in vascular senescence and disease. The review describes the microvascular dysfunction as a precursor that emerges early in aging, and as a marker that predicts cardiovascular risk associated with an increase in oxidative stress and a decrease in nitric oxide (NO), which drive endothelial cell senescence. Chronic low-grade inflammation (“inflammaging”) dysregulates homeostasis and leads to age-related CVD disease. The review highlights the role of oxidative stress in the mitochondrial respiration process and the production of reactive oxygen species (ROS), which drive immune cell dysfunction and epigenetic changes. More specifically, the authors described the process of chronic inflammation that uncouples endothelial nitric oxide synthase (eNOS) and ROS generation promoted by NADPH oxidase (Nox), leading to vascular damage and activation of cell senescence and immune signaling pathways (NF-κB/NLRP3), triggering vascular inflammation through pro-inflammatory cytokine release. Activation of NLRP3, NF-κB inflammatory pathways, oxidative stress and endothelial dysfunction are promoted by trimethylamine N-oxide (TMAO) during dysbiosis. However, short-chain fatty acids (SCFAs) reduce inflammation and arterial stiffness, and their decrease is usually correlated with vascular aging. Kasal et al., emphasized this in potential strategies to mitigate end-organ damage and age-related vascular injury.

Vascular remodeling, stiffness and dysfunction are hallmarks of the pulmonary artery (PA) in the elderly, leading to vascular damage and associated diseases, which include pulmonary arterial hypertension (PAH). To measure PA stiffness, PA global longitudinal strain (GLS) and non-contrast cardiovascular magnetic resonance (CMR) are the least invasive methods compared to invasive echocardiography or hemodynamics, which are mainly used for disease cohorts. To potentially enable early detection and targeted therapies for pulmonary vascular disease and to identify metabolic pathways underlying PA stiffness, Zhang et al., investigated PA stiffness using an approach linking serum metabolomics with PA GLS. Vascular damage is associated with metabolic disorders identified by circulating metabolites such as lipids, oxidative stress markers or amino acids. These molecular fingerprints are used as metabolic predictors of vascular injury.

In a prospective study using a cohort of 170 elderly patients, Zhang et al., found that CMR and PA GLS were associated with cardiovascular risk and aging. Furthermore, they suggested the implication of collagen synthesis, fatty acid oxidation and gluconeogenesis as metabolic pathways involved in PA stiffness, and that PA GLS may serve as a new tool to identify early metabolic dysregulation related to pulmonary dysfunction in asymptomatic elderly individuals.

Metabolic dysfunction in the elderly is usually associated with imbalances in oxidative stress pathways, leading to complications of both systemic and cardiovascular injury. Dietary antioxidant intake, as evaluated by the Composite Dietary Antioxidant Index (CDAI), has shown a potential beneficial effect on inflammation and oxidative stress associated with diabetes and obesity. However, there is no clear evidence on its effect on the prevalence of CVD in the elderly. Run Wang et al. investigated the association of CDAI with cardiovascular disease in adults. They analyzed data from 25,997 adults from NHANES (2011–2020). These revealed a negative association between CDAI and CVD prevalence. In their study, they highlighted gender-specific protective effects, suggesting a reduction in CVD risk, particularly in women. The study is original, however, and to confirm their findings, prospective studies are needed to confirm the causality and to unravel the signaling pathways involved.

We would like to express our profound gratitude to all the authors who have contributed to this Research Topic by submitting their best works.

